# Breast Cancer Systemic Treatments and Upper Limb Lymphedema: A Risk-Assessment Platform Encompassing Tumor-Specific Pathological Features Reveals the Potential Role of Trastuzumab

**DOI:** 10.3390/jcm8020138

**Published:** 2019-01-24

**Authors:** Marco Invernizzi, Anna Michelotti, Marianna Noale, Gianluca Lopez, Letterio Runza, Massimo Giroda, Luca Despini, Concetta Blundo, Stefania Maggi, Donatella Gambini, Nicola Fusco

**Affiliations:** 1 Physical and Rehabilitative Medicine, Department of Health Sciences, University of Eastern Piedmont “A. Avogadro”, Viale Piazza D’Armi 1, 28100 Novara, Italy; marco.invernizzi@med.uniupo.it; 2 Division of Pathology, Fondazione IRCCS Ca’ Granda, Ospedale Maggiore Policlinico, Via Francesco Sforza 35, 20122 Milan, Italy; anna.michelotti@studenti.unimi.it (A.M.); gianluca.lopez@unimi.it (G.L.); letterio.runza@policlinico.mi.it (L.R.); 3 Division of Medical Oncology, Fondazione IRCCS Ca’ Granda, Ospedale Maggiore Policlinico, Via Francesco Sforza 35, 20122 Milan, Italy; donatella.gambini@policlinico.mi.it; 4 National Research Council (CNR), Neuroscience Institute Aging Branch, Via Giustiniani 2, 35128 Padua, Italy; marianna.noale@in.cnr.it (M.N.); stefania.maggi@in.cnr.it (S.M.); 5 School of Pathology, University of Milan, Via Festa del Perdono 7, 20122 Milano, Italy; 6 Division of Breast Surgery, Fondazione IRCCS Ca’ Granda, Ospedale Maggiore Policlinico, Via Francesco Sforza 35, 20122 Milan, Italy; massimo.giroda@policlinico.mi.it (M.G.); luca.despini@policlinico.mi.it (L.D.); concetta.blundo@policlinico.mi.it (C.B.); 7 Department of Biomedical, Surgical and Dental Sciences, University of Milan, Via Della Commenda 10, 20122 Milan, Italy

**Keywords:** breast cancer related lymphedema, breast cancer, lymphovascular invasion, extranodal extension, therapy, axillary lymph nodes dissection, radiation therapy, chemotherapy, taxanes, trastuzumab, anti-HER2

## Abstract

Breast cancer related lymphedema (BCRL) is frequent but strategies for an individualized risk assessment are lacking. We aimed to define whether tumor-specific pathological features, coupled with clinical and therapeutic data, could help identify patients at risk. Data from 368 patients with node-positive breast cancers were retrospectively collected, including 75 patients with BCRL (0.4–25.6 years follow-up). BCRL was assessed during the standard follow-up oncology visits using the circumferential measurement. Clinicopathologic and therapeutic factors associated with BCRL were integrated into a Cox proportional hazards regression model. Lymphovascular invasion (LVI) was more common in BCRL patients (*n* = 33, 44% vs. *n* = 85, 29%, *p* = 0.01), akin extra nodal extension (ENE) of the metastasis (*n* = 57, 76% vs. *n* = 180, 61%, *p* = 0.02). Sentinel lymph node excision without axillary dissection and extra-axillary radiotherapy were BCRL-unrelated. A higher number of BCRL-positive patients were treated with taxane-based chemotherapy with or without trastuzumab, compared to BCRL-negative patients (*p* < 0.01). Treatment with trastuzumab and/or taxanes, adjusted for systemic infections, laterality, therapy, and pathological features (i.e., LVI and ENE), had a significant impact in BCRL-free survival (*p* < 0.01). This work offers new insights on BCRL risk stratification, where the integration of clinical, therapeutic, and tumor-specific pathological data suggests a possible role of anti-human epidermal growth factor receptor 2 (HER2) therapy in BCRL pathogenesis.

## 1. Background

Treatment of breast cancer has progressed extraordinarily with the introduction of more and more personalized therapies [1]. However, the improvement in survival observed in recent decades led to an increased incidence of long-term complications related to the treatments [2,3,4,5]. Among them, breast cancer related lymphedema (BCRL) is one of the most frequent issues in breast cancer survivors treated with surgery with or without radiotherapy, and has tremendous implications on women’s quality of life and on sanitary costs [6,7]. This condition is due to an iatrogenic impairment in the transport capacity of the local lymphatic system after surgery and radiotherapy, which leads to the interstitial accumulation of lymph fluid in the upper limb [8]. BCRL has a progressive clinical course, usually showing suboptimal response to surgical, physical, and medical therapies [9]. There are several lines of evidence to suggest that the early detection of BCRL is cornerstone to allow for an effective treatment. However, the preventive options available to date are scarce. As a consequence, complete remission in these patients is seldom accomplished [7].

All patients subjected to axillary procedures (e.g., en bloc dissection, sentinel lymph node, and irradiation) should be considered at risk for BCRL [6,10,11,12,13]. The number of lymph nodes excised plays a crucial role, not only for mechanical reasons but probably also because of the increased use of radiotherapy in patients with numerous metastatic nodes [10,13,14,15]. Furthermore, patients subjected to radical mastectomy show a higher frequency of BCRL compared to those treated with breast-conserving surgery (i.e., quadrantectomy, lumpectomy) [16,17]. Some authors have also hypothesized the role of taxane-based chemotherapy in BCRL pathogenesis, but only in women subjected to axillary lymph nodes dissection (ALND) [18,19,20,21]. Other risk factors include high body mass index (BMI), smoking, and alcoholism, confirming the role of non-communicable diseases in long-term complications in breast cancer survivors [22]. There is recent evidence that tumor-specific biological features, such as peritumoral lymphovascular invasion (LVI) and extranodal extension (ENE), are involved in BCRL pathogenesis [14]. Regrettably, all these variables are not integrated in BCRL clinical workup.

Realizing new strategies for assessing the individual risk of lymphedema in breast cancer survivors is a crucial clinical need. Our working hypothesis was that the integration of tumor-specific biological features with patients’ clinical and therapeutic data could improve BCRL risk stratification. Using an integrative risk-assessment platform, we sought to characterize new risk factors for BCRL.

## 2. Materials and Methods

### 2.1. Study Cohort

This observational study included a retrospective series of women with node-positive (pN ≥ 1) breast cancers diagnosed from January 1998 to September 2018 at Fondazione IRCCS Ca’ Granda Ospedale Maggiore Policlinico, Milan, Italy. All patients underwent breast surgery with standard adjuvant treatment (medical and radiotherapy) that was based on risk stratification. Outcome data were collected, and only patients with uniformly recorded BCRL status and detailed information on the adjuvant therapeutic protocols employed were included. Likely or documented syndromic tumors, pregnancy-associated breast cancers, or those treated in the neoadjuvant setting were excluded for this study. Patients were anonymized prior to data collection and analysis. The study was approved by the local Independent Ethics Committee under protocol number #620_2018bis.

### 2.2. BCRL Assessment

For all patients, BCRL was assessed during the standard follow-up oncology visits using the circumferential method [14,23]. In the presence of macroscopic evidence and/or patients complains of BCRL (e.g., swelling, heaviness, pain, aching, limb fatigue, impaired mobility), the limb volume was calculated considering the arm either as a single segment or as the sum of the truncated cone volumes represented by the multiple segments. For this analysis, sequential circumferential measurements were taken every 5-cm interval, distally and proximally to the crease of the elbow, using a tape and compared to the contralateral arm. In the presence of an interlimb difference ≥2 cm at any single location or ≥200 mL, BCRL was confirmed and annotated as a dichotomous variable (i.e., BCRL+ and BCRL−) [24].

### 2.3. Histological Review 

Tumor classification, grading, and pathological staging were re-performed for all cases following the latest recommendations [25] and guidelines [26,27]. All cases were independently reviewed by three pathologists with a particular interest in breast pathology (Gianluca Lopez, Letterio Runza, and Nicola Fusco). Discordant results were resolved collegially. LVI and ENE were assessed following the College of American Pathologists 2017 Protocol for the examination of specimens from patients with invasive carcinoma of the breast (v.4.0.0.0, available at https://www.cap.org/cancerprotocols), as previously described [14,28,29,30,31,32,33,34].

### 2.4. Statistical Analyses

All the statistical analyses were performed using the SAS 9.4 software (SAS Institute, Cary, NC, USA). Categorical variables were annotated as absolute numbers with the corresponding percentage. To summarize the continuous variables, the mean and standard deviation (SD) or median and quartiles (Q1, Q3) were used. The Shapiro-Wilk test was employed to analyze the normal distributions. Associations between BCRL, demographic and clinical information, pathologic and molecular features of the tumors, and treatment data were performed using the Fisher’s exact test, chi-squared test, or Wilcoxon rank-sum, according to the type of variables. Subsequently, Cox’s proportional hazard regression models were used for multivariate analyses, and covariates were selected using the purposeful method [14,35]. The proportional hazard assumption was verified considering Schoenfeld’s residuals of each covariate [36]. The linearity assumption for quantitative variables was evaluated on the basis of the quartiles [37]. For each predictor, the hazard ratio (HR) and 95% confidence intervals (CI) were provided. Survival curves were built according to the Kaplan-Meier method and compared using the Log-Rank test, as previously described [38]. All statistical tests were two-tailed; only *p*-values of less than 0.05 were considered statistically significant.

## 3. Results

A total of 368 women (age, 26–88 years; median, 58 years) with pN ≥ 1 breast cancer were included in this study (follow-up time, 0.4–25 years; median 6.2 years). Among them, 75 (20%) patients were BCRL-positive and 293 (80%) were BCRL-negative. The median time of BCRL onset was 1.6 years (range 0.2–8.2 years). The demographic data and general characteristics of the study group are listed in Table 1.

### 3.1. Tumor-Specific Biological Features Associated with BCRL

Lymphedema was observed more frequently in patients with cancers of the right breast (*n* = 48, 64%, *p* = 0.02), whereas in the BCRL-negative population the tumors were equally distributed among the left (*n* = 151, 51%) and right (*n* = 142, 49%) sides. In both cohorts the most frequently diagnosed histological type was the invasive ductal carcinoma. No statistically significant differences among BCRL-positive and BCRL-negative tumors were observed in terms of tumor stage, histological grade, proliferation index, and hormone receptor status. LVI at the periphery of the primary tumor was detected in 44% (*n* = 33) of BCRL patients, while only 29% (*n* = 85) of the BCRL-negative population showed this feature (*p* = 0.01). The prevalence of ENE of the metastasis was significantly higher (*p* = 0.02) in BCRL-positive (*n* = 57, 76%) than in BCRL-negative patients (*n* = 180, 61%). These observations confirm that intrinsic biological features of both the tumor and metastasis are bona fide biomarkers of BCRL occurrence. All clinicopathologic features are summarized in Supplementary Table S1.

### 3.2. The Type of Axillary Surgical Dissection But Not the Type of Breast Surgery Impacts on BCRL

The condition of BCRL was restricted to the patients subjected to ALND, as those who underwent only sentinel lymph node procedure (*n* = 19, 7%) were all BCRL-negative (*p* = 0.02). Breast conservative surgery was the most widely adopted surgical approach both in BCRL-positive (*n* = 47, 63%) and BCRL-negative (*n* = 177, 60%) patients, as shown in Table 2. No statistically significant correlation was observed between the type of breast surgery and BCRL. These data provide circumstantial evidence that BCRL is likely not to be a direct consequence of the breast surgery and that non-invasive procedures in the axilla are not able alone to trigger this condition.

### 3.3. Extra-Axillary Radiotherapy Does Not Increase the Risk of BCRL

Whole breast irradiation (WBI) was the most frequently adopted radiotherapy protocol among both BCRL-positive (*n* = 43, 57%) and BCRL-negative (*n* = 169, 58%) patients, including 7 (9%) and 16 (6%) cases, respectively, subjected to additional irradiation of the supraclavicular fossa (Table 2). WBI was performed after surgery as one treatment per day, five days a week, for five to seven weeks. A supplemental boost dose has been variably included at the end of the regimen. In our cohort, all patients treated with mastectomy and radical lymphadenectomy showing ≥4 metastatic lymph nodes received radiotherapy on both the supraclavicular fossa and the chest wall (*n* = 57, 16%). None of the patients included in this study received axillary irradiation, while 21 (28%) BCRL-positive and 78 (27%) BCRL-negative patients were radiotherapy-naïve. Overall, extra-axillary radiation therapy was not associated with BCRL (Table 2).

### 3.4. Taxanes-Based Chemotherapy Is Associated with BCRL Occurrence

**Figure 1:**
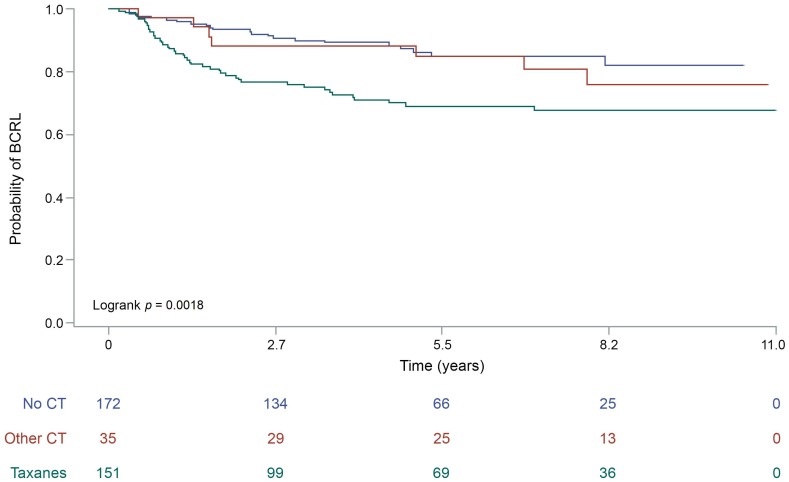
Lymphedema-free survival of the patients included in the study according to the type of chemotherapy. The curves are built according to the by Kaplan-Meier method, *p* values are the expression of Log-rank test. The specific risk for a given timeframe is reported on the bottom of each graph. BCRL, breast cancer related lymphedema; CT, chemotherapy.

Fisher’s exact test showed a strong association between adjuvant chemotherapy and BCRL (*p* < 0.01), as displayed in Table 2. Taken together, a higher prevalence of chemo-treated patients was observed in the BCRL-positive cohort (*n* = 53, 71%), compared to the BCRL-negative (*n* = 140, 48%), as shown in Table 2. In particular, 45 (60%) BCRL patients were treated with taxane-based protocols (e.g., epirubicin and cyclophosphamide followed by docetaxel), in contrast to BCRL-negative patients (*n* = 110, 38%). The higher incidence of BCRL was not observed in patients treated with other (i.e., taxanes-free) protocols (Table 2). These results were corroborated by the observation that more than half of unaffected subjects (*n* = 153, 52%) received neither taxanes nor other chemotherapy protocols. Log-rank test demonstrated that taxanes, but not other chemotherapeutics, had a significant impact on BCRL-free survival (*p* < 0.01), as represented in Figure 1. The association between chemotherapy and BCRL was further evaluated using a Cox proportional hazard model (Table 3). This analysis showed that taxanes administration exposes breast cancer patients to a doubled risk of developing lymphedema (HR = 2.24, 95% CI (1.26–3.98)), compared to other protocols (Table 3). No significant correlations were observed between BCRL and hormone therapy, regardless of both the therapeutic protocol and duration of the treatment (Table 2).

### 3.5. Anti-Human Epidermal Growth Factor Receptor 2 (HER2) Monoclonal Antibodies Administration Increase the Risk of BCRL

**Figure 2:**
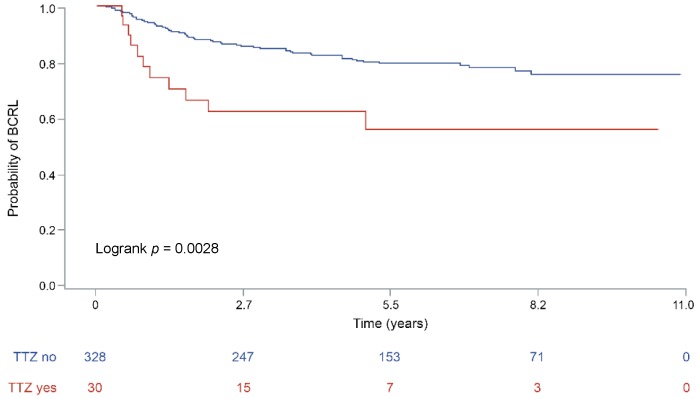
Lymphedema-free survival of the patients included in the study according to the administration of anti-human epidermal growth factor receptor 2 (HER2) therapy. The curves are built according to the by Kaplan-Meier method, *p* values are the expression of Log-rank test. The specific risk for a given timeframe is reported on the bottom of each graph. BCRL, breast cancer related lymphedema; TTZ, trastuzumab.

**Figure 3:**
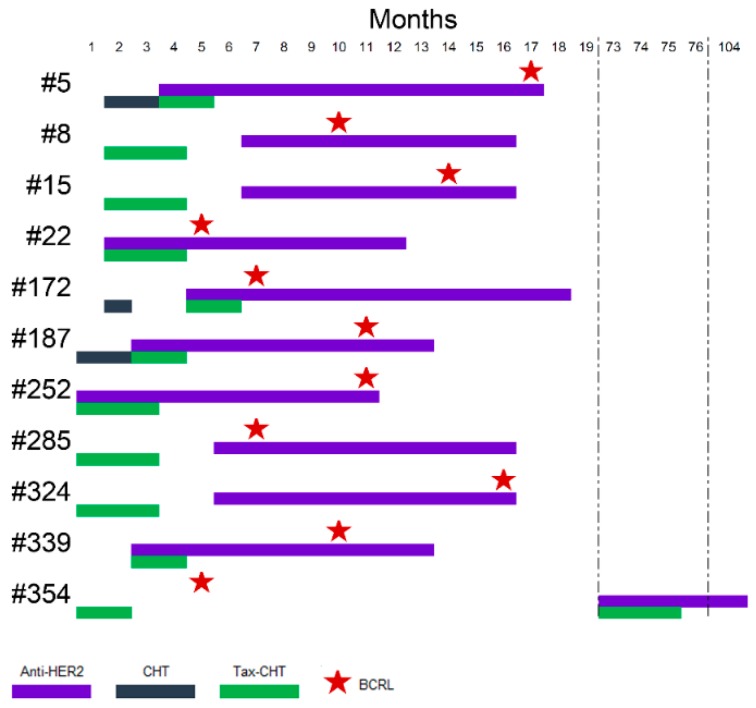
Schematic representation of the therapeutic history of BCRL patient. The timeline depicts the months after surgery, as reported on the top; patients are reported as rows, according to their ID on the left; the type of therapy is color-coded on the basis of the legend on the bottom. The first BCRL diagnosis is highlighted as a red star in the timeline. CHT, chemotherapy (no taxanes); Tax-CHT, taxane-based chemotherapy; BCRL, breast cancer related lymphedema.

The rate of human epidermal growth factor receptor 2 (HER2)-positive carcinomas was higher in patients with BCRL (*n* = 11, 15%) than in BCRL-negative (*n* = 26, 9%), albeit not statistically significant (Supplementary Table S1). All HER2-positive patients with BCRL, before developing lymphedema, were treated with the monoclonal antibody trastuzumab (TTZ), while only 19 (73%) women with HER2-positive tumors (*n* = 26) in the BCRL-negative group were treated with anti-HER2 therapy. According to Fisher’s exact test, the association between anti-HER2 targeted therapy and BCRL was significant, (*p* = 0.02), as shown in Table 3. This association was retained while performing survival analyses (*p* < 0.01), as depicted in Figure 2. Despite the small sample size, the significance was maintained after the inclusion of TTZ therapy in the Cox Proportional Hazard model comprising LVI and ENE (*p* = 0.01). This analysis provided evidence that anti-HER2 therapy with TTZ increased the risk of subsequent BCRL by nearly three times (HR = 2.7, 95% CI (1.31–5.55)). The therapeutic timeline of BCRL patient treated with anti-HER2 drugs is outlined in Figure 3.

## 4. Discussion

BCRL occurs in a remarkable proportion of breast cancer survivors, thus involving hundreds of thousands of women worldwide, with a significant impact on social and sanitary costs [7]. Despite risk factors have been identified, patients risk stratification is not performed to date. As a result, BCRL is treated at its occurrence, in contrast with the modern principles of preventive medicine. Regrettably, BCRL is poorly responsive to surgical, physical, and medical therapies. Therefore, the currently available preventive strategies, if applied at an individualized level, could improve patients’ outcome and sanitary interventions. We have hypothesized that the integration of multiple clinical information with tumor-specific pathological information would be germane for improving the currently available guidelines to define individuals at risk. Here, we performed a comprehensive analysis of a large series of surgically-treated pN ≥ 1 breast cancers with long-term follow-up and found that extra-axillary radiotherapy, sentinel lymph node procedure, and extensive surgical procedures on the breast are not able alone to increase the risk of BCRL. Furthermore, we confirm the strong predictive value of LVI and ENE for BCRL occurrence and that their evaluation is able to identify new risk factors, such as TTZ therapy. Finally, we corroborate the notion that, among chemo-treated patients, BCRL occurs preferentially in the setting of therapy with taxanes.

In our study, we provide new insights on how breast cancer systemic therapy can influence BCRL-free survival. The potential association of endocrine therapy, chemotherapy and anti-HER2 targeted therapy with lymphedema was evaluated with a comprehensive risk model, encompassing both treatment-related and pathological indicators. Data of our cohort of patients confirmed the detrimental impact of adjuvant chemotherapy. In detail, HR significantly increases when patients are treated with taxanes-based protocols, supporting previous literature findings [20,21]. In initial stages, lymphedema development is characterized by interstitial fluid retention and accumulation of protein-rich fluid. This process may be worsened by adjuvant chemotherapy with a mechanism that closely resembles early pathological changes typical of secondary lymphedema. Indeed, taxanes, and in particular docetaxel, can lead to an enhancement of interstitial fluid filtration, capillary protein leakage and subsequently edema [39]. Therefore, adjuvant taxanes-based protocols can contribute to the initiation and maintenance of BCRL by increasing fluid collection early on after surgery, when lymphatic drainage is markedly impaired by axillary dissection procedures. 

More interestingly, we were able to identify a significant correlation between anti-HER2 therapy and BCRL. Despite the small sample size of the HER2-positive tumors included in this study, our data provides previously unavailable evidence that patients who receive TTZ are at higher risk of postsurgical lymphedema. Given that the adjuvant systemic therapy with TTZ requires the administration of concurrent chemotherapy, we can hypothesize the additive effect of anti-HER2 monoclonal antibodies to chemotherapeutics. To date, no adverse effects of TTZ on microcirculation or fluid retention have been described. However, this monoclonal antibody selectively binds HER2, which is part of human epidermal growth factor receptor family [40]. HER2 overexpression has been linked to higher levels of vascular endothelial growth factor (VEGF)-C, which is a key player in lymphatic development, and subsequent increase in lymphangiogenesis [41]. Evidences in vitro showed that VEGF-C mRNA and protein expression decreased significantly in breast cancer cells after TTZ, supporting the assumption of a clinically relevant association between HER2 and VEGF-C levels [42]. Therefore, the blockade of HER2 by monoclonal antibodies, such as TTZ, may on one hand reduce direct proliferative effects of VEGF-C on tumor cells and on the other hand, for its systemic effect, diminish lymphangiogenesis in the site of surgery, preventing lymphatic regeneration and thus promoting lymphedema.

This study has intrinsic limitations. First, its retrospective nature prevented a rigorous and uniform measurement of the arm volume. Hence, the sensitivity of the circumferential method has been questioned by many authors, particularly in the presence of arm shape irregularities and gibbousness [43,44]. Overall, the lack of a baseline measurement of the limb volume prior to the development of macroscopic BCRL, coupled with the fact that the measurements were not always taken at regular intervals, could have led to an underestimation of the real incidence of BCRL in our population of patients. To overcome potential biases in quantification we chose to annotate BCRL as a dichotomous variable. However, this study should be considered hypothesis-generating. Further clinical studies coupled with detailed records of both arm volumes at regular intervals using cutting-edge tools (i.e., bioimpedance spectroscopy or tissue dielectric constant analysis), information on weight gain and symptoms of fluid retention (e.g., facial edema) during chemotherapy, and duration of the specific interventions will be required to allow for BCRL staging, including latent and subclinical conditions. Second, the relatively small sample size of HER2-positive tumors included in the study could have limited the clinical impact of our conclusions. It should be noted, however, that our work provides for the first time in literature evidence on the possible role of trastuzumab in BCRL pathogenesis. Large-scale studies enriched for HER2-positive breast cancers are warranted to confirm our observations. Third, we cannot rule out the possibility that the correlation of TTZ treatment in BCRL onset could be related to a carry-over effect of the combined treatment with taxanes, in particular considering the different protocols adopted during the long time-frame of patients’ recruitment (1993–2018). To this end, functional studies exploring the individual role of specific drugs in BCRL would be needed.

## 5. Conclusions

Despite these limitations, our work offers novel insights in BCRL risk stratification, where the integration of demographic, clinical, therapeutic, and tumor-specific pathologic data could represent a step forward for the prevention, or at least prediction, of long-term complications in breast cancer survivors. The assessment of LVI and ENE could be beneficial not only for the implementation of BCRL active surveillance protocols in the setting of trastuzumab therapy, but also for tailoring the surgical intervention, follow-up, and therapeutic strategy in women with node-positive breast cancer.

## Figures and Tables

**Table 1:** Demographic data and general characteristics of the patients included in the study. BCRL, breast cancer related lymphedema; SD, standard deviation; BMI, body mass index.

	BCRL+ (*n* = 75)	BCRL− (*n* = 293)	*p*-Value
Age at diagnosis, years, mean ± SD	57.9 ± 13.1	59.5 ± 12.9	0.3879
Menopause, *n* (%)			
Peri-	1 (1.4)	9 (3.1)	0.6845
Post-	49 (67.1)	195 (67.7)	
Pre-	23 (31.5)	84 (29.1)	
Smoking status, current smoker, *n* (%)	8 (10.7)	29 (9.9)	0.8433
BMI, mean ± SD	27.0 ± 5.6	26.9 ± 5.4	0.9572
Obesity, BMI ≥ 30 kg/m^2^ *n* (%)	16 (21.3)	78 (26.6)	0.3488
Diabetes mellitus, *n* (%)	7 (9.3)	19 (6.5)	0.3903
Cardiovascular diseases, *n* (%)	25 (33.3)	83 (28.3)	0.3956
Systemic infections, *n* (%)	10 (13.3)	18 (6.1)	0.0361
Blood disorders, *n* (%)	6 (8.0)	25 (8.5)	0.8822
Bone and joints diseases, *n* (%)	7 (9.3)	32 (10.9)	0.6901
Dyslipidemia, *n* (%)	5 (6.7)	44 (15.0)	0.0575
Gastrointestinal diseases, *n* (%)	14 (18.7)	51 (17.4)	0.7984
Diseases of the urinary tract, *n* (%)	4 (5.3)	16 (5.5)	1.0000
Diseases of the reproductive tract, *n* (%)	12 (16.0)	49 (16.7)	0.8805
Central nervous system diseases, *n* (%)	1 (1.3)	25 (8.5)	0.0299
Other neoplasms, *n* (%)	10 (13.3)	42 (14.3)	0.8242

**Table 2:** Therapeutic protocols of the patients included in the study.

	BCRL+ (*n* = 75)	BCRL− (*n* = 293)	*p*-Value
Breast surgery, *n* (%)			
Conservative	47 (62.7)	177 (60.4)	0.7208
Mastectomy	28 (37.3)	116 (39.6)	
Axillary surgery, *n* (%)			
En bloc dissection	75 (100.0)	274 (93.5)	0.0178
Sentinel lymph node	0 (0.0)	19 (6.5)	
Radiotherapy, *n* (%)			
No WBI WBI + SCF SCF + CW	21 (28.0) 36 (48.0) 7 (9.3) 11 (14.7)	78 (26.6) 153 (52.2) 16 (5.5) 46 (15.7)	0.6318
Chemotherapy, *n* (%)			
No Taxanes Other protocols	22 (29.3) 45 (60.0) 8 (10.7)	153 (52.2) 110 (37.5) 30 (10.2)	0.0010
Hormone therapy, *n* (%)			
No SERM Aromatase inhibitors SERM + Aromatase inhibitors SERM + LHRH agonists	14 (18.7) 6 (8.0) 34 (45.3) 11 (14.7) 10 (13.3)	36 (12.3) 43 (14.7) 135 (46.1) 44 (15.0) 35 (12.0)	0.4206
Duration of the intake (days), median (Q1, Q3)			
SERM Aromatase inhibitors LHRH agonists	301 (252; 349) 1586 (1095; 1827) 1200 (731; 1358)	300 (216; 371) 1544 (967; 1836) 989 (708; 1765)	1.0000 0.8658 0.9343
Trastuzumab, *n* (%)	11 (14.7)	19 (6.5)	0.0209
Breast surgery, *n* (%)			
Conservative	47 (62.7)	177 (60.4)	0.7208
Mastectomy	28 (37.3)	116 (39.6)	
Axillary surgery, *n* (%)			
En bloc dissection	75 (100.0)	274 (93.5)	0.0178
Sentinel lymph node	0 (0.0)	19 (6.5)	
Radiotherapy, *n* (%)			
No WBI WBI + SCF SCF + CW	21 (28.0) 36 (48.0) 7 (9.3) 11 (14.7)	78 (26.6) 153 (52.2) 16 (5.5) 46 (15.7)	0.6318
Chemotherapy, *n* (%)			
No Taxanes Other protocols	22 (29.3) 45 (60.0) 8 (10.7)	153 (52.2) 110 (37.5) 30 (10.2)	0.0010
Hormone therapy, *n* (%)			
No SERM Aromatase inhibitors SERM + Aromatase inhibitors SERM + LHRH agonists	14 (18.7) 6 (8.0) 34 (45.3) 11 (14.7) 10 (13.3)	36 (12.3) 43 (14.7) 135 (46.1) 44 (15.0) 35 (12.0)	0.4206
Duration of the intake (days), median (Q1, Q3)			
SERM Aromatase inhibitors LHRH agonists	301 (252; 349) 1586 (1095; 1827) 1200 (731; 1358)	300 (216; 371) 1544 (967; 1836) 989 (708; 1765)	1.0000 0.8658 0.9343
Trastuzumab, *n* (%)	11 (14.7)	19 (6.5)	0.0209

BCRL, breast cancer related lymphedema; WBI, whole breast irradiation; SCF, supraclavicular fossa; CW, chest wall; SERM, selective estrogen receptor modulator (Tamoxifen); LHRH, luteinizing hormone releasing hormone agonist; Q1, quartile 1; Q3, quartile 3.

**Table 3:** Clinicopathologic factors associated with the development of breast cancer related lymphedema (Cox proportional hazard model).

		HR	95% CI	*p*-Value
Systemic infections		1.88	0.95–3.71	0.0703
Chemotherapy, no		1.00		
Taxanes Other protocols		2.24 1.21	1.26–3.98 0.50–2.94	0.0060 0.6684
Trastuzumab		2.70	1.31–5.55	0.0071
No radiotherapy, no		1.00		
WBI WBI + SCF SCF + CW		0.73 0.71 0.45	0.42–1.28 0.29–1.73 0.20–0.98	0.2678 0.4549 0.0446
Side	ENE			0.0144
Right	Yes vs. No	3.11	1.45–6.65	
Left	Yes vs. No	0.76	0.32–1.78	
Side	LVI			0.0208
Right	Yes vs. No	1.09	0.59–2.00	
Left	Yes vs. No	3.56	1.61–7.87	

HR, hazard ratio; CI, confidence interval; WBI, whole breast irradiation; SCF, supraclavicular fossa; CW, chest wall; ENE, extranodal extension; LVI, lymphovascular invasion.

## Supplementary Materials

The following are available online at http://www.mdpi.com/2077-0383/8/2/138/s1, Table S1: Clinicopathologic features of the tumors.
