# Supplementary material for: Breast Cancer Systemic Treatments and Upper Limb Lymphedema: A Risk-Assessment Platform Encompassing Tumor-Specific Pathological Features Reveals the Potential Role of Trastuzumab
Source: J Clin Med. 2019 Jan 24;8(2):138. doi: 10.3390/jcm8020138 (PMC6406664; doi:10.3390/jcm8020138)
Supplement: Supplementary file 1 [file jcm-08-00138-s001.pdf]

Supplementary

Table S1. Clinicopathologic features of the tumors. BCRL, breast cancer related lymphedema; ER, estrogen receptor; PR, progesterone receptor; LN, lymph nodes.

	**BCRL+ (*n* = 75)**	**BCRL− (*n* = 293)**	***p*-Value**
Side, right *n* (%)	48 (64.0)	142 (48.6)	0.0163
Histotype, *n* (%)			0.0964
No special type (ductal)	67 (89.3)	238 (81.2)	
Other	8 (10.7)	55 (18.8)	
pT, *n* (%)			0.8501
T1	43 (57.3)	168 (57.3)	
T2	25 (33.3)	91 (31.1)	
T3	3 (4.0)	10 (3.4)	
T4	4 (5.3)	24 (8.2)	
pN, *n* (%)			0.1405
N1	40 (53.3)	192 (65.5)	
N2	18 (24.0)	55 (18.8)	
N3	17 (22.7)	46(15.7)	
Grade, *n* (%)			0.4122
1	3 (4.00)	25 (8.5)	
2	40 (53.3)	146 (49.8)	
3	32 (42.7)	122 (41.6)	
ER+, *n* (%)	64 (85.3)	263 (89.8)	0.2768
PR+, *n* (%)	58 (77.3)	242 (82.6)	0.2949
HER2+, *n* (%)	11 (14.7)	26 (8.9)	0.1366
Ki67-high, *n* (%)	36 (48.0)	120 (41.0)	0.2707
Receptor status, *n* (%)			0.1265
ER+, HER2-	56 (74.7)	248 (84.6)	
HER2+	11 (14.7)	26 (8.9)	
ER-, HER2-	8 (10.7)	19 (6.5)	
Lymphovascular invasion, *n* (%)	33 (44.0)	85 (29.0)	0.0131
Extranodal extension, *n* (%)	57 (76.0)	180 (61.4)	0.0187
Nr. metastatic LN, median (Q1; Q3)	3 (1; 7)	2 (1; 5)	0.0434
Total nr. of LN, median (Q1; Q3)	23 (18; 29)	23 (19; 30)	0.4854
% metastatic LN, median (Q1; Q3)	13 (6; 30)	10 (5; 25)	0.2573
